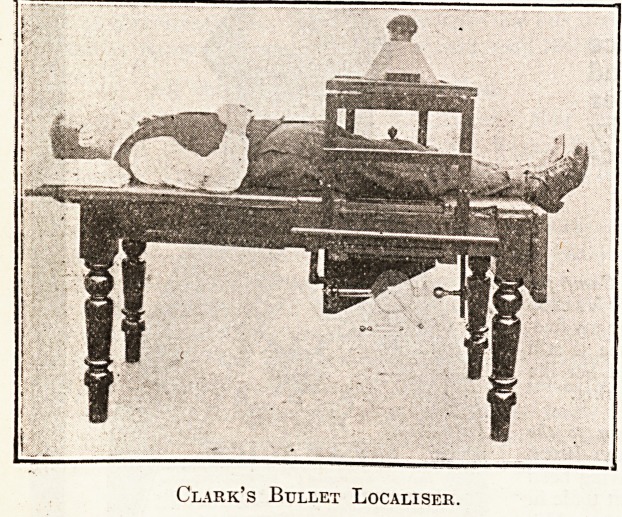# Clark's Fluorescent Screen Bullet Localiser

**Published:** 1915-01-09

**Authors:** 


### Clark's Fluorescent Screen Bullet Localiser.

WE have been asked to draw attention to this apparatus as having a special field of usefulness in the present circumstances. Its purpose is the rapid and accurate localisation of bullets when these have entered any part of the body and are retained in the tissues. The details of construction and arrangement are, of course, highly technical, but it is sufficient to say that by an ingenious method and in succession two shadows can be rapidly obtained, and by plotting out the results on a special indicator the exact position of the foreign body can be determined. We have reason to believe that the method is a good one, but, as practical surgeons know, it is one thing to localise a bullet and another thing to reach and remove it. What seems, and indeed is, perfectly precise in a picture becomes much less simple amidst the anatomical disturbances of a surgical operation. Clark's apparatus was demonstrated to the Röntgen Ray Society last month, and one of them is being fitted up at the 3rd London General Hospital at Wandsworth. We may therefore expect to hear reports of its value as tested in the field of surgical practice, and we shall be glad to publish such reports when they come to hand. Put shortly, Clark's apparatus may perhaps be described as a simplification of the old Mackenzie-Davidson method.

CLARK'S BULLET LOCALISER.

The inventor, Mr. Clark, c/o T. Clark and Son, Falmouth Road, Bishopston, Bristol, gives the following practical details of the method :

1. To mark the body front and back (with iodine or a patch of adhesive plaster) to indicate where the vertical *x*-rays enter and leave the body; into this path the bullet can be easily brought, as shown upon the fluorescent screen at the intersection of cross-wires.

2. To obtain the depth of the bullet the tube is moved across the body 4 inches, and the shadow of the bullet then falls into a different position upon the screen. Provision is made to indicate this second position, and the amount of movement of the shadow can be measured after the screen is removed and the light turned up.

3. The position of the upper cross-wires can be marked on the upper aspect of the body by means of a plumb-bob suspended from the cross-wire, also on the lower aspect of the body from the wires below the table.

The results can be readily plotted out on a patent indicator. The fixed and known distances are shown on the indicator, the variable distances being shown afterwards by the movement of a lever and cross-thread over a scaled rule, which indicates the depth of the foreign body below the mark on the upper aspect of the body.

## Figures and Tables

**Figure f1:**